# Correction: Two Intense Decades of 19^th^ Century Whaling Precipitated Rapid Decline of Right Whales around New Zealand and East Australia

**DOI:** 10.1371/journal.pone.0096729

**Published:** 2014-04-25

**Authors:** The PLOS ONE Staff

## Notice of Republication

This article was republished on April 11, 2014, to correct an error that marked coauthor Tim D. Smith as deceased. The publisher apologizes for this error. Please download this article again to view the correct version. The originally published, uncorrected article and the republished, corrected article are provided here for reference.

## Supporting Information

File S1 Originally published, uncorrected article.
(PDF)

File S2 Republished corrected article.
(PDF)
