# Supplementary material for: Two Intense Decades of 19th Century Whaling Precipitated Rapid Decline of Right Whales around New Zealand and East Australia
Source: PLoS One. 2014 Apr 1;9(4):e93789. doi: 10.1371/journal.pone.0093789 (PMC3972245; doi:10.1371/journal.pone.0093789)
Supplement: Sampling Protocol S1 — Methods for obtaining American logbook data for New Zealand calving bays. (DOC) [file pone.0093789.s001.doc]

Supplemental Material “Sampling Protocol S1”

**Methods for obtaining American logbook data for New Zealand calving bays**

We attempted to identify all American whaling vessels that were bay whaling in each year from three published sources. First we used the American Offshore Whaling Voyage (AOWV) data to identify all voyages that had listed New Zealand as a destination on customs forms at the beginning of the voyage and were at sea from 1834 to 1841. Second we examined McNab to identify vessels in calving bays in each winter. Finally we used Langdon , who had used American logbooks to identify vessels that made port for refitting and repairs in proximity to New Zealand and eastern Australia each winter, and hence could have been in a position to have been bay whaling.

For each of those candidate vessels in each year we examined available information on vessel location and season, cross-referenced by those ships reported in bays by McNab to determine how likely it was that each did in fact engage in bay whaling. We scored each vessel in each year as (1) highly likely to have been bay whaling, (2) possibly to have been bay whaling, and (3) very unlikely to have been bay whaling. We estimated the number of vessel-seasons of bay whaling as being between the number highly likely bay whaling and that number plus the number possibly bay whaling (Sampling Protocol Table S1).

Of the vessels that were highly likely to have been bay whaling, we identified extant voyage logbooks using Lund et al , and sampled some of these. For each legible logbook that covered the entire winter, we recorded the number of right whales struck and landed and those struck but lost reported for each year. The logbook often included reports of whales killed by whalers from other vessels that were working together, and where possible we included only whales processed by the vessel creating the logbook; however at times the distinction was not possible. In one case the logbook appeared to report all whales taken by any of three cooperating vessels. We also relied on total seasonal oil production reported occasionally by McNabb and estimated whales landed by dividing those values by 40 barrels per whale (see shore-based whaling methods above). We estimate struck but lost rates for bay whaling using data from the logbooks read, using the same methods as for the ship-based offshore whaling, above.

| Sampling Protocol Table S1. List of vessels highly likely (High) and possibly (Poss) bay whaling in New Zealand by year, showing vessel number (VN) and voyage identification number (VID) (after Lund et al ). | | | | |
| --- | --- | --- | --- | --- |
| Vessel | VN | VID | Year | Likely |
| Erie | 1315 | 4588 | 1834 | High |
| Warren | 2578 | 15340 | 1835 | High |
| Benjamin Rush | 971 | 1768 | 1836 | High |
| Columbus | 1124 | 3059 | 1836 | High |
| Erie | 1315 | 4590 | 1836 | High |
| Favourite | 228 | 4904 | 1836 | High |
| Franklin | 1405 | 5209 | 1836 | High |
| Friendship | 1420 | 5330 | 1836 | High |
| Gratitude | 248 | 6003 | 1836 | High |
| Jasper | 330 | 7313 | 1836 | High |
| John Adams | 1704 | 7596 | 1836 | High |
| Martha | 401 | 9148 | 1836 | High |
| Mary Mitchell | 1893 | 9384 | 1836 | High |
| Navy | 495 | 10341 | 1836 | High |
| Nile | 491 | 10487 | 1836 | High |
| Samuel Robertson | 606 | 12807 | 1836 | High |
| Sarah Lee | 2350 | 12927 | 1836 | Poss |
| South Boston | 2407 | 13272 | 1836 | High |
| Tuscaloosa | 652 | 14152 | 1836 | High |
| Vermont | 2555 | 15119 | 1836 | High |
| Warren | 2578 | 15340 | 1836 | High |
| Bowditch | 1001 | 1970 | 1837 | Poss |
| Chariot | 1068 | 2572 | 1837 | High |
| Courier | 99 | 3448 | 1837 | High |
| Erie | 1315 | 4590 | 1837 | High |
| Gratitude | 248 | 6003 | 1837 | High |
| Huntress | 277 | 6887 | 1837 | Poss |
| Julian | 323 | 7930 | 1837 | High |
| Martha | 401 | 9148 | 1837 | High |
| Mary Mitchell | 1893 | 9384 | 1837 | Poss |
| Orozimbo | 511 | 11000 | 1837 | High |
| Pantheon | 2144 | 11246 | 1837 | High |
| Rosalie | 2318 | 12533 | 1837 | High |
| Thule | 2500 | 13967 | 1837 | High |
| Tuscaloosa | 652 | 14152 | 1837 | High |
| Virginia | 669 | 15192 | 1837 | High |
| William Thompson | 683 | 15738 | 1837 | Poss |
| Adeline | 2 | 252 | 1838 | High |
| Alexander Barclay | 59 | 512 | 1838 | High |
| Almira | 806 | 666 | 1838 | Poss |
| Averick | 11 | 1452 | 1838 | High |
| Averick Heinecken | 773 | 1455 | 1838 | Poss |
| Bowditch | 1001 | 1970 | 1838 | High |
| Chariot | 1068 | 2572 | 1838 | High |
| Columbus | 1124 | 3060 | 1838 | High |
| Erie | 1315 | 4591 | 1838 | High |
| Fortune | 224 | 5049 | 1838 | High |
| Friendship | 1420 | 5331 | 1838 | High |
| Gold Hunter | 1479 | 5840 | 1838 | High |
| Gratitude | 248 | 6004 | 1838 | Poss |
| Houqua | 275 | 6809 | 1838 | High |
| Hydaspe | 269 | 6928 | 1838 | Poss |
| Izette | 1668 | 7205 | 1838 | High |
| Jasper | 1691 | 7414 | 1838 | Poss |
| Java | 317 | 7432 | 1838 | Poss |
| John Adams | 320 | 7605 | 1838 | Poss |
| Julian | 323 | 7930 | 1838 | High |
| Lucy Ann | 1814 | 8676 | 1838 | High |
| Luminary | 1819 | 8681 | 1838 | Poss |
| Merrimack | 1935 | 9655 | 1838 | Poss |
| Montano | 1965 | 9972 | 1838 | High |
| Rajah | 576 | 12104 | 1838 | High |
| Rosalie | 2318 | 12533 | 1838 | High |
| Sarah Frances | 2347 | 12920 | 1838 | High |
| Shylock | 2390 | 13173 | 1838 | High |
| Thomas Dickason | 657 | 13803 | 1838 | High |
| Vermont | 2555 | 15120 | 1838 | High |
| Warren | 2578 | 15341 | 1838 | High |
| Addison | 3 | 224 | 1839 | Poss |
| Adeline | 2 | 252 | 1839 | High |
| Amethyst | 8 | 875 | 1839 | High |
| Arab | 899 | 1156 | 1839 | High |
| Atlantic | 925 | 1366 | 1839 | High |
| Cherokee | 101 | 2805 | 1839 | High |
| China | 92 | 2846 | 1839 | High |
| Erie | 1315 | 4591 | 1839 | High |
| Falcon | 211 | 4827 | 1839 | Poss |
| Favourite | 228 | 4906 | 1839 | High |
| General Williams | 1445 | 5526 | 1839 | High |
| George | 1465 | 5550 | 1839 | High |
| Grand Turk | 260 | 5999 | 1839 | High |
| Gratitude | 248 | 6004 | 1839 | High |
| Helvetia | 1568 | 6368 | 1839 | High |
| Izette | 1668 | 7205 | 1839 | High |
| Jasper | 1691 | 7414 | 1839 | High |
| John and Edward | 325 | 7648 | 1839 | High |
| Julius Caesar | 1737 | 7946 | 1839 | High |
| Luminary | 1819 | 8681 | 1839 | High |
| Lydia | 1825 | 8728 | 1839 | High |
| Magellan | 1828 | 8756 | 1839 | High |
| Martha | 1869 | 9090 | 1839 | Poss |
| Mary | 1895 | 9171 | 1839 | Poss |
| Merrimac | 1935 | 9655 | 1839 | Poss |
| N. P. Talmadge | 2020 | 10291 | 1839 | High |
| Navy | 495 | 10342 | 1839 | High |
| Newton | 493 | 10452 | 1839 | Poss |
| North America | 2058 | 10611 | 1839 | High |
| Parachute | 539 | 11261 | 1839 | Poss |
| Roman | 580 | 12477 | 1839 | High |
| Samuel Robertson | 606 | 12808 | 1839 | High |
| Sarah Frances | 2347 | 12920 | 1839 | High |
| Shylock | 2390 | 13174 | 1839 | Poss |
| South Boston | 2407 | 13273 | 1839 | High |
| Superior | 2448 | 13556 | 1839 | High |
| Thorn | 2496 | 13911 | 1839 | High |
| Warren | 2578 | 15341 | 1839 | High |
| Washington | 686 | 15386 | 1839 | Poss |
| White Oak | 2601 | 15493 | 1839 | High |
| Alexander Barclay | 59 | 512 | 1840 | High |
| Ann Maria | 879 | 1032 | 1840 | Poss |
| Bowditch | 1001 | 1971 | 1840 | Poss |
| Canova | 1030 | 2285 | 1840 | Poss |
| Chariot | 1068 | 2573 | 1840 | High |
| Cherokee | 101 | 2805 | 1840 | High |
| Clematis | 1112 | 2959 | 1840 | Poss |
| Columbia | 1122 | 3046 | 1840 | High |
| Columbus | 1124 | 3061 | 1840 | High |
| Cora | 100 | 3312 | 1840 | Poss |
| Erie | 1315 | 4591 | 1840 | High |
| Factor | 221 | 4794 | 1840 | Poss |
| Fanny | 1361 | 4895 | 1840 | High |
| Favourite | 228 | 4906 | 1840 | High |
| Franklin | 219 | 5219 | 1840 | Poss |
| Friends | 1418 | 5319 | 1840 | Poss |
| General Williams | 1445 | 5526 | 1840 | High |
| Georgia | 1468 | 5753 | 1840 | Poss |
| Golconda II | 245 | 5832 | 1840 | Poss |
| Gold Hunter | 1479 | 5841 | 1840 | Poss |
| Good Return | 218 | 5899 | 1840 | High |
| Hamilton | 1515 | 6094 | 1840 | Poss |
| Harvest | 1551 | 6234 | 1840 | Poss |
| Hercules | 271 | 6538 | 1840 | Poss |
| Heroine | 1597 | 6614 | 1840 | High |
| Janus | 322 | 7372 | 1840 | Poss |
| Jasper | 1691 | 7415 | 1840 | Poss |
| Java | 317 | 7433 | 1840 | Poss |
| John Wells | 333 | 7819 | 1840 | High |
| Julian | 323 | 7931 | 1840 | Poss |
| Lucy Ann | 1814 | 8677 | 1840 | Poss |
| Marcia | 459 | 8872 | 1840 | Poss |
| Martha | 1869 | 9090 | 1840 | Poss |
| Mayflower | 418 | 9476 | 1840 | Poss |
| Montpelier | 421 | 10053 | 1840 | Poss |
| Neptune | 2032 | 10371 | 1840 | Poss |
| Newton | 493 | 10452 | 1840 | High |
| Octavia | 502 | 10742 | 1840 | Poss |
| Portland | 2222 | 11870 | 1840 | Poss |
| Rajah | 576 | 12106 | 1840 | Poss |
| Rosalie | 2318 | 12534 | 1840 | Poss |
| Selma | 605 | 13117 | 1840 | Poss |
| South Boston | 2407 | 13274 | 1840 | High |
| Superior | 2448 | 13556 | 1840 | Poss |
| Thomas Dickason | 657 | 13804 | 1840 | High |
| Tuscarora | 2518 | 14165 | 1840 | Poss |
| Vermont | 2555 | 15121 | 1840 | High |
| American | 867 | 860 | 1841 | High |
| China | 92 | 2847 | 1841 | Poss |
| Falcon | 211 | 4828 | 1841 | Poss |
| Lucas | 375 | 8631 | 1841 | High |
| Parachute | 539 | 11262 | 1841 | Poss |
| Phenix | 2180 | 11550 | 1841 | Poss |
| Roman II | 580 | 12478 | 1841 | Poss |

Supplementary Protocol References

1. Lund JN, Josephson E, Reeves RR, Smith T (2010) American Offshore Whaling Voyages: 1667 – 1927. Volume I: Voyages by Vessel; Volume II: Voyages by Master. Old Dartmouth Historical Society – New Bedford Whaling Museum, New Bedford Massachusetts.

2. McNab R (1913) The old whaling days: A history of southern New Zealand from 1830 to 1840. Christchurch: Whitcombe & Tombs.

3. Langdon R (1984) Where the whalers went: an index to the Pacific ports and islands visited by American whalers, and some other ships, in the 19th century. Canberra: Pacific Manuscripts Bureau. 298 p.
