# Supplementary material for: Two Intense Decades of 19th Century Whaling Precipitated Rapid Decline of Right Whales around New Zealand and East Australia
Source: PLoS One. 2014 Apr 1;9(4):e93789. doi: 10.1371/journal.pone.0093789 (PMC3972245; doi:10.1371/journal.pone.0093789)
Supplement: Table S1 — Estimated shore-based whaling catches, listed as number of southern right whales, with primary source listed in column to right of catch for catches at New South Wales that were from New Zealand (NSW-NZ) and two estimated coastal catch series for New Zealand (NZ-Low, NZ-High), reflecting different selections of primary sources. (DOCX) [file pone.0093789.s002.docx]

Table S1: Estimated shore-based whaling catches, listed as number of southern right whales, with primary source listed in column to right of catch for catches at New South Wales that were from New Zealand (NSW-NZ) and two estimated coastal catch series for New Zealand (NZ-Low, NZ-High), reflecting different selections of primary sources.

| **Year** | **NSW-NZ** | **Source** | **NZ Low** | **SE** | **Source** | **NZ High** | **SE** | **Source** |
| --- | --- | --- | --- | --- | --- | --- | --- | --- |
| 1829 | 0 | D |  |  |  | 24 |  | M |
| 1830 | 0 | D | 28 |  | GBP1 | 120 |  | M |
| 1831 | 0 | D | 30 |  | GBP1 | 239 |  | M |
| 1832 | 0 | D | 23 |  | GBP1 | 140 |  | M |
| 1833 | 62 | D | 56 |  | GBP1 | 295 |  | M |
| 1834 | 118 | D | 84 |  | GBP1 | 333 |  | M |
| 1835 | 271 | D | 98 |  | GBP1 | 446 |  | M |
| 1836 | 127 | D | 82 |  | GBP1 | 341 |  | M |
| 1837 | 198 | D | 72 |  | GBP1 | 226 |  | M |
| 1838 | 325 | D | 145 |  | GBP1 | 440 |  | M |
| 1839 | 390 | D | 128 |  | GBP1 | 158 |  | GBP1 |
| 1840 | 242 | D | 86 |  | GBP1 | 143 |  | GBP1 |
| 1841 | 166 | D | 57 |  | GBP1 | 95 |  | GBP1 |
| 1842 | 249 | D | 25 |  | GBP1 | 61 |  | GBP1 |
| 1843 | 50 | D | 332 |  | BB | 332 |  | BB |
| 1844 | 85 | D | 276 |  | BB | 276 |  | BB |
| 1845 | 66 | D | 187 |  | GBP2 | 187 |  | GBP2 |
| 1846 | 54 | D | 151 |  | GBP2 | 151 |  | GBP2 |
| 1847 | 41 | D | 134 |  | BB | 134 |  | BB |
| 1848 | 23 | D | 83 |  | BB | 83 |  | BB |
| 1849 | 10 | D | 27 |  | BB | 27 |  | BB |
| 1850 | 15 | D | 17 |  | BB | 17 |  | BB |
| 1851 | 10 | D | 5 |  | BB | 5 |  | BB |
| 1852 | 21 | D | 17 |  | BB | 17 |  | BB |
| 1853 | 34 | D | 14 |  | BB | 31 |  | SNZ - Oil |
| 1854 | 1 | D | 13 |  | SNZ-Oil | 13 |  | SNZ - Oil |
| 1855 | 7 | D | 22 |  | SNZ-Oil | 22 |  | SNZ - Oil |
| 1856 | 10 | D | 34 |  | SNZ-Oil | 34 |  | SNZ - Oil |
| 1857 | 0 | D | 28 |  | SNZ-Oil | 28 |  | SNZ - Oil |
| 1858 | 0 | D | 13 |  | SNZ-Oil | 13 |  | SNZ - Oil |
| 1859 | 17 | D | 22 |  | SNZ-Oil | 22 |  | SNZ - Oil |
| 1860 | 11 | D | 2 |  | SNZ-Oil | 2 |  | SNZ - Oil |
| 1861 | 5 | D | 2 |  | SNZ-Oil | 11 |  | SNZ- Baleen |
| 1862 | 5 | D | 7 |  | SNZ-Oil | 9 |  | SNZ- Baleen |
| 1863 | 9 | D | 5 |  | SNZ- Baleen | 33 |  | SNZ-Oil |
| 1864 | 7 | D | 3 | 4 | SNZ- Baleen | 17 |  | SNZ-Oil |
| 1865 | 7 | D | 1 |  | SNZ- Baleen | 12 | 14 | SNZ-Oil |
| 1866 | 0 | D | 1 | 1 | SNZ- Baleen | 7 | 7 | SNZ-Oil |
| 1867 | 1 | D | 1 |  | SNZ- Baleen | 7 |  | SNZ-Oil |
| 1868 | 1 | D | 4 | 5 | SNZ- Baleen | 9 |  | SNZ-Oil |
| 1869 | 0 | D | 9 |  | SNZ- Baleen | 21 |  | SNZ-Oil |
| 1870 | 0 | D | 10 |  | SNZ- Baleen | 23 |  | SNZ-Oil |
| 1871- 1900 | 0 | D | 226 |  | SNZ-Oil, Baleen | 356 |  | SNZ-Oil, Baleen |
| 1901-1930 | 0 | D | 143 |  | SNZ-Oil, Baleen | 143 |  | SNZ-Oil, Baleen |
| Total | 2638 |  | 2703 |  |  | 5104 |  |  |

Sources are: D: Dawbin [17]; M: McNab [30]; GBP: Great Britain Parliamentary papers, (1) is [24] and (2) is [25]; SNZ: Statistics New Zealand [31]; BB: Blue Books [26].
