# Supplementary material for: Correction: Two Intense Decades of 19th Century Whaling Precipitated Rapid Decline of Right Whales around New Zealand and East Australia
Source: PLoS One. 2014 Apr 25;9(4):e96729. doi: 10.1371/journal.pone.0096729 (PMC4000189; doi:10.1371/journal.pone.0096729)
Supplement: File S1 — Originally published, uncorrected article. (PDF) [file pone.0096729.s001.pdf]

PLOS ONE

Two Intense Decades of 19th Century Whaling Precipitated Rapid Decline of Right Whales around New Zealand and East Australia

Emma L. Carroll[1*¤], **Jennifer A. Jackson**[2], **David Paton**[3], **Tim D. Smith**[4†]

1 School of Biological Sciences, University of Auckland, Auckland, New Zealand, 2 British Antarctic Survey, Cambridge, Cambridgeshire, United Kingdom, 3 Blue Planet Marine, Canberra, Australian Capital Territory, Australia, 4 World Whaling History, Redding, California, United States of America

Abstract

Right whales (*Eubalaena* spp.) were the focus of worldwide whaling activities from the 16th to the 20th century. During the first part of the 19th century, the southern right whale (*E. australis*) was heavily exploited on whaling grounds around New Zealand (NZ) and east Australia (EA). Here we build upon previous estimates of the total catch of NZ and EA right whales by improving and combining estimates from four different fisheries. Two fisheries have previously been considered: shore-based whaling in bays and ship-based whaling offshore. These were both improved by comparison with primary sources and the American offshore whaling catch record was improved by using a sample of logbooks to produce a more accurate catch record in terms of location and species composition. Two fisheries had not been previously integrated into the NZ and EA catch series: ship-based whaling in bays and whaling in the 20th century. To investigate the previously unaddressed problem of offshore whalers operating in bays, we identified a subset of vessels likely to be operating in bays and read available extant logbooks. This allowed us to estimate the total likely catch from bay-whaling by offshore whalers from the number of vessels seasons and whales killed per season: it ranged from 2,989 to 4,652 whales. The revised total estimate of 53,000 to 58,000 southern right whales killed is a considerable increase on the previous estimate of 26,000, partly because it applies fishery-specific estimates of struck and loss rates. Over 80% of kills were taken between 1830 and 1849, indicating a brief and intensive fishery that resulted in the commercial extinction of southern right whales in NZ and EA in just two decades. This conforms to the global trend of increasingly intense and destructive southern right whale fisheries over time.

Citation: Carroll EL, Jackson JA, Paton D, Smith TD (2014) Two Intense Decades of 19th Century Whaling Precipitated Rapid Decline of Right Whales around New Zealand and East Australia. PLoS ONE 9(4): e93789. doi:10.1371/journal.pone.0093789

Editor: Athanassios C. Tsikliras, Aristotle University of Thessaloniki, Greece

Received October 20, 2013; **Accepted** March 7, 2014; **Published** April 1, 2014

Copyright: © 2014 Carroll et al. This is an open-access article distributed under the terms of the Creative Commons Attribution License, which permits unrestricted use, distribution, and reproduction in any medium, provided the original author and source are credited.

Funding: Funding for the review and report preparation was provided by the New Zealand Ministry of Fisheries through Project ZBD200505 to the National Institute of Water and Atmospheric Research (NIWA), Oregon State University General Research Fund to JJ and CS Baker and the Lenfest Ocean Program of the Pew Charitable Trust to SR Palumbi. EC was supported by a fellowship from the Tertiary Education Commission and TS through the History of Marine Animal Populations (HMAP) project. The funders had no role in study design, data collection and analysis, decision to publish, or preparation of the manuscript.

Competing Interests: The authors have the following interests: David Paton is employed by Blue Planet Marine. This does not alter the authors' adherence to PLOS ONE policies on sharing data and materials.

* E-mail: elc6@st-andrews.ac.uk

¤ Current address: Scottish Oceans Institute, University of St Andrews, St Andrews, Fife, Scotland

†Deceased.

Introduction

Right whales (*Eubalaena spp*) were a primary target of whalers from the mid 16th century to the late 20th century [1]. Pursued for oil, processed from their thick blubber, and for their baleen, all three species (North Pacific: *E. japonica*; North Atlantic: *E. glacialis* and southern: *E. australis*) were greatly reduced in abundance in all oceans by a series of, at times, short-term fisheries [2].

Today, the North Pacific and North Atlantic species are represented by small, remnant populations occupying a fraction of their historical range [3–5]. In contrast, the southern right whale shows spatially variable recovery: some populations are recovering at close to the biological maximum rate (e.g. South Africa [6]) whereas others are possibly functionally extinct (e.g. Chile [7]). Extant populations of southern right whales show significant differences in maternally-inherited mitochondrial DNA haplotype frequencies, consistent with female fidelity to migratory destinations [8]. This behaviourally-mediated mechanism of isolation, coupled with the spatially fragmented recovery, suggests each population underwent its own decline due to whaling and subsequent recovery (or lack thereof). Therefore, historical assessments should be attempted at a population level, to provide accurate historical records of past abundances, distribution and catch histories.

Here we focus on reconstructing the catch history for southern right whales around New Zealand and east Australia. The historical patterns of seasonal migration, distribution and abundance of southern right whales in this region are complex and not well understood. Historical records suggest there were two distinct coastal whaling grounds around New Zealand islands: New Zealand sub-Antarctic (Auckland and Campbell Islands) and mainland New Zealand (North and South Islands) [9]. Mainland New Zealand was a coastal calving ground where females would give birth in the bays and inlets during winter. It is unclear whether the New Zealand sub-Antarctic was historically a calving or feeding ground, or a mixture of both [10]. The two areas could

have been linked by large-scale migration patterns, as the species moved offshore during the austral summer to feed [10]. The New Zealand sub-Antarctic is now the primary calving ground for the New Zealand population [11]. Although historically severely depleted, the New Zealand population now numbers 2,000 whales and is growing at around 7% per annum, based on a mark-recapture study [12]. The two areas appear to be inhabited by one contemporary population based on genetic studies and the movements of individuals [11,13], although it is unclear whether this was true historically.

Across the southern coast of Australia, wintering aggregations of southern right whales, particularly cows with calves, were found prior to whaling [2]. The species moved offshore during the austral summer, presumably migrating to feeding grounds. Although there is little evidence to suggest subdivision of calving grounds from the historical data [2], today there is a clear difference in recovery between southern right whales in southwest and southeast Australia. The southwest population is growing at 6.8% per annum and numbers approximately 3,000 whales [14]. In contrast, the southeast Australian population numbers approximately 500 whales, and it does not appear to be recovering at the same speed as the southwest [15].

Investigations of current population structure show there are significant differences in maternally-inherited mitochondrial DNA haplotype frequencies and bi-parentally inherited nuclear microsatellite markers between southern right whales in southwest Australia and New Zealand, indicating some degree of isolation between these two populations [13]. Preliminary findings, based on a small sample from the southeast Australia population, indicates this is a small, remnant population, distinct from New Zealand and southwest Australia. However, there is only weak genetic differentiation between the southeast Australian and New Zealand populations, suggesting the two populations could have current or historic gene flow [13]. Additionally, the two populations could have mixed during offshore migration, for example, in the Tasman Sea or during migrations to feeding grounds [16]. Therefore, there are biological reasons to consider both regions when reconstructing catch histories in the region.

In a global perspective describing shore-based and offshore whaling, seven whaling operations taking right whales around eastern Australia and New Zealand were identified [1]. Shore-based whaling began around 1805 in bays around Tasmania and the Australian mainland, and later developed in bays in New Zealand [1]. Pelagic or ship-based offshore whaling was pursued in the eastern Australian and New Zealand region by Australian and New Zealand vessels and by French, British and American registered vessels from the 1820s [1]. This was part of a much broader global whale fishery involving primarily right and sperm whales [1].

The earliest evidence of whaling in New Zealand and eastern Australian waters are reports of a 1791 port call by a whaling vessel in New Zealand and some 1805 shore whaling activity near Hobart [17]. Although little information on the magnitude of right whale catches is available for the earliest years, information on the catches of the seven whaling operations noted above is available from several primary sources beginning in 1827 [1]. One source is tabulations of returns of fisheries and the related, but less easily interpreted, records of whale oil and baleen exports, both kept by colonial or national authorities. Export records do not reflect whale oil used locally and do not necessarily relate to the year of capture.

These data were reported in barrels of oil, and usually distinguished between the more valuable oil from sperm whales (sperm oil) and the less valuable oil from right whales ("whale oil" or "black oil"). This latter term was also used for oil from pinnipeds. The reports of oil and baleen can be converted to rough numbers of whales using average numbers of barrels and pounds of baleen obtained per whale, respectively, for a subset of logbooks where those quantities were recorded for individuals or small groups of whales [17].

A second source of catch data is lists of whaling voyages, such as those tabulated for example by Starbuck [18] for American whaling vessels and by Du Pasquier [19] for French whaling vessels. These lists vary in completeness and in the information tabulated. A third source of data is daily logbooks kept by ship-based whalers, which frequently include information on numbers and species of whales captured, numbers struck and lost, and locations where whales were sought and where they were sighted or caught. Finally, there is published information about 20th century right whaling in this region, including illegal Soviet whaling [20]. Dawbin [17] estimated southern right whale catches by both shore-based and ship-based whaling using some of these sources of data. Here we review and extend his estimates using the same sources and additional sources not previously available to improve the historical catch series for New Zealand and east Australian right whales.

We consider two scenarios in order to capture the biological and historical uncertainties in the catch series: the strict New Zealand catch series, and the catch series for New Zealand plus east Australia. Vessels from Hobart and Sydney went bay whaling in New Zealand and sometimes the catch was itemised, allowing New Zealand catches to be correctly assigned to the country of origin [17,21]. This is not always the case, meaning some New Zealand catches were incorrectly assigned to the east Australian shore-based catch series. Combining the totals from colonies in southeast Australia and New Zealand should provide a complete catch series that is representative of the overall region [17,21]. In addition, the weak level of genetic differentiation between the New Zealand and east Australian stocks seen today could be due to recent divergence and the regions could have been historically linked by gene flow, with some New Zealand whales available for capture in east Australian waters and vice versa. It should be noted that the historical records available do not permit a comprehensive catch series for southwest Australia to be reconstructed [22].

We account for the distribution of catches over time for four fisheries: shore-based whaling in bays, ship-based whaling in bays, ship-based whaling offshore and whaling in the 20th century. We improve upon previous work by multiplying our estimates of catches by new, fishery-specific estimates of struck and lost whales to obtain estimates of total removals, and include explicit estimates of sampling uncertainties for some of these fisheries. These estimates of total removals of right whales are designed for use in the modelling of right whale population history in this region and represent a significant expansion on previous work, resulting in more comprehensive estimates of total catch and removals that allow insights into the development of the fishery over time.

Materials and Methods

Shore-based Whaling

Our reconstruction of the shore-based catches is based on different data for New Zealand and east Australia. In both cases, the primary reference sources were export records and returns of fisheries, as described in Dawbin [17] and in a reference bibliography [23]. For the Australian catches, we did not review the primary sources due to limitations in access and resources available, but rather used the coastal catch series presented by Dawbin [17].

Table 1. Estimated coastal catches from shore-based whaling operations, listed as number of southern right whales, for Victoria (VIC), Tasmania (TAS), New South Wales (NSW), and the catches at New South Wales that were from New Zealand (NSW-NZ) are reproduced from Dawbin [17] for convenience.

Year	VIC	TAS	NSW	NSW-NZ	NZ-Low	NZ-High
1827	0	64	0	0	0	0
1828	0	109	10	0	0	0
1829	0	131	9	0	0	24
1830	0	233	103	0	28	120
1831	0	195	201	0	30	239
1832	0	246	49	0	23	140
1833	0	346	94	62	56	295
1834	61	356	237	118	84	333
1835	170	409	279	271	98	446
1836	97	493	235	127	82	341
1837	142	815	401	198	72	226
1838	3	844	435	325	145	440
1839	60	1064	539	390	128	158
1840	0	804	17	242	86	143
1841	44	279	198	166	57	95
1842	5	167	320	249	25	61
1843	27	277	58	50	332	332
1844	35	241	114	85	276	276
1845	4	259	91	66	187	187
1846	21	85	140	54	151	151
1847	8	104	60	41	134	134
1848	3	70	77	23	83	83
1849	1	24	34	10	27	27
1850	1	46	76	15	17	17
1851	1	32	129	10	5	5
1852	0	13	24	21	17	17
1853	0	8	78	34	14	31
1854	0	0	27	1	13	13
1855	0	0	10	7	22	22
1856	0	0	23	10	34	34
1857	0	0	11	0	28	28
1858	0	0	5	0	13	13
1859	0	0	52	17	22	22
1860	0	0	42	11	2	2
1861	0	0	24	5	2	11
1862	0	0	57	5	7	9
1863	0	0	11	9	5	33
1864	0	0	22	7	3	17
1865	0	0	15	7	1	12
1866	0	0	12	0	1	7
1867	0	2	30	1	1	7
1868	0	6	45	1	4	9
1869	0	2	10	0	9	21
1870	0	1	28	0	10	23
1871–1900	0	20	136	0	226	356
1901–1930	0	0	7	0	143	143
**Total**	**683**	**7,745**	**4,575**	**2,638**	**2,703**	**5,104**

Table 1. Cont.

Two estimated coastal catch series for New Zealand (NZ-Low, NZ-High), reflecting different selections of primary sources as described in Table S1.
doi:10.1371/journal.pone.0093789.t001

For New Zealand shore-based catches, the amount of whale oil and/or baleen reported was tallied for each year from 1829 for each primary source used by Dawbin [17]: Great Britain Parliamentary Records [24–25], Blue book of statistics from the Great Britain Colonial Office [26], McNab [27–30], Sherrin [21], Statistics New Zealand [31] and Wakefield [32]. To convert the oil and baleen data reported in those sources to numbers of whales, one whale was estimated to have produced 4.18 tuns of black oil or 600 pounds of baleen, based on the average yield from 413 shore-caught whales in Dawbin [17]. Rarely, the number of whales caught was available for specific years, and where available these values were used rather than values based on reported oil and baleen. The catch series reconstructed from each primary source was then compared with the catch history in Dawbin [17] and the original source reference that he used for each year was identified by this comparison. Some errors in the original catch series were identified using primary sources, and catch estimates were modified accordingly (Table 1).

For the years 1853–1930, the catch series was based on Statistics New Zealand records of baleen and right whale (black) oil exports. For each series, the amount exported each year was tallied and missing data were interpolated using a five-year moving average and the variance around this average was calculated. The export series was then converted into whales using the above conversion rates.

Ship-based Offshore Whaling

Ship-based offshore whaling and bay whaling in Australia and New Zealand was undertaken by French and American ships, and apparently to a lesser extent by British, Australian and New Zealand vessels [1]. Dawbin [17] estimated catch by French and American ships, and we first sought to reconstruct those estimates using his primary data sources. We then expanded upon that previous work on American ship-based whaling by reading a selection of logbooks that were relatively complete for location and species hunted data and by explicitly including uncertainty in our estimates.

Dawbin [17] estimated the landed catch of right whales in the vicinity of New Zealand by 19th century American and French whalers using information contained in lists of whaling voyages. For American whalers, Starbuck [18] listed summary information for individual whaling voyages including departure and subsequent arrival dates back to their home ports, customs forms entries of intended destination, and total landings of sperm and baleen products such as oil and baleen. For French whalers, Du Pasquier [19] listed similar summary information for individual whaling voyages, but also included information on the locations visited during the voyage and for many voyages the numbers of sperm and right whales taken. Dawbin [17] used these two data sources to estimate annual catches in both of the American and the French ship-based fisheries operating both offshore and in New Zealand bays.

For both US and French whalers, Dawbin identified from the two voyage lists those vessels thought to have whaled in New

Zealand or Australia [17]. For American voyages, this included those that indicated in customs forms that they were bound for the New Zealand area [18]. For French voyages, this included voyages reported to have been in New Zealand or Australian waters [19]. In both cases, all whales or whale oil reported landed from each identified voyage was assumed to be from right whales from the New Zealand area. Using the American Offshore Whaling Voyage data (see below: AOWV) [33] and our own digitization of Du Pasquier's [19] data, we were able to reproduce the estimates of both US and French right whale catches for all voyages departing in a given year [17].

We sought to improve on Dawbin's [17] estimates of catches of American whalers by using two new sets of data: the AOWV dataset [33], and the American Offshore Whaling Logbook (AOWL) dataset [34]. The AOWV dataset includes one record for each of the roughly 15,000 multi-year American whaling voyages known to have occurred from 1667 to 1927. Data recorded include voyage dates, vessel details, and amount of sperm and baleen whale oil ultimately landed. The AOWL dataset includes one record for each day at sea of a sample of roughly 10% of the American whaling voyages represented in the AOWV. The AOWL data include information on vessel tracks, whales encountered, whales killed, and at times volume of oil obtained from individual whales, all extracted from original logbooks kept by the whalers at sea.

We omitted data for some of the voyages in the AOWL sample to account for various irregularities in the original logbooks and in the completeness of the information extracted. The logbooks used to obtain these data varied in their completeness, with varying proportions of the whales identified to species, with varying proportions of voyage days being reported, and with sometimes continuous gaps in reporting. To minimize bias due to these problems, we selected logbooks for voyages where at least 75% of the whales taken were identified to species, where there was information for more than 70% of the days and where the gaps between daily entries were fewer than 10% of the total number of recorded days. Further, we only selected voyage logbooks that reported sufficient right whales to account for the whale oil reported for the voyage in the AOWV data, assuming the average barrels of whale oil per whale where oil yields for individual whales had been identified (81 barrels per right whale, SE 3.8 from AOWL data where barrels reported).

We selected AOWL data within the study area boundaries 140°E (including east Australia, which we consider to be New South Wales, Victoria and Tasmania) to 140°W and approximately 27°S to 60°S (Figure 1). Finally, we excluded voyages from the AOWV data and from the AOWL data that were of less than 16 months duration or that returned only sperm oil because in the AOWL sample such voyages did not take right whales in the study area.

These selection criteria resulted in AOWL data from 280 logbooks. We combined the AOWL and the AOWV data to estimate the total number of right whales taken by American voyages in the study areas as follows. We computed the number of voyages departing in each year (i) from the AOWV data (N_i), the number of voyages departing in each year from the AOWL data (n_i), the number of the latter that reported whaling at sometime during the voyage in New Zealand or east Australian waters (m_i), and the number of right whales caught while they were in those waters (r_i). We estimated the catches of right whale in these waters for each year of departure i as:

$$N_i\left(\frac{m_i}{n_i}\right)\left(\frac{r_i}{m_i}\right) \quad (1)$$

That is, the estimated removals are the product of the total number of voyages departing, the fraction of those that whaled in the study area, and the mean catch of right whales by those voyages from the study area. This simplifies to:

$$N_i\left(\frac{r_i}{n_i}\right), \quad (2)$$

the number of voyages departing multiplied by the mean catch of right whales in New Zealand, or east Australian, waters by all sampled voyages. The variance of these estimates is based on the variance of the takes of right whales per voyage among the sample voyages.

We then attempted to accurately reflect the year of actual take by whaling vessels. For each departure year, we estimated the number of visits to the New Zealand area by vessels in each whaling season (September to May) following a vessel's departure from home ports in the North Atlantic accounting for the time required to travel to the New Zealand area. We treated the number of visits as multinomial random variables and estimated the mean proportion in each year and the corresponding variances accordingly, ignoring the covariances. As there was no trend in the estimated proportions over departure years, we combined the data and used the aggregate proportions to allocate the estimates of total takes by vessels departing in a given year to the seasons of actual take.

British, Australian and later New Zealand whalers also operated in the New Zealand region but Dawbin had no information on them that would allow him to estimate their right whale catches. We examined information from port arrival records of whaling vessels in the Bay of Islands [35] on the type of oil obtained, but were also unable to estimate catches by these vessels.

Ship-based Bay Whaling

A substantial number of American offshore whaling vessels were known to take southern right whales while in bays in the winter where the species calved, operating alongside the shore-based whalers described above [30]. Examination of AOWL data for some of the voyages identified by McNab [30] revealed temporal gaps in the AOWL data for winter months, resulting in a lack of data on catches in the calving bays. This appears to have been a limitation of the sampling protocol used for some of the data. In the end, there were too few data on catches in New Zealand bays in the AOWL data to allow this component of the fishery to be adequately represented. Thus our estimates for catches by American ship-based whaling described above apply only when those ships were operating offshore. To estimate the catches when American ships were operating in New Zealand bays, we identified American voyages that likely engaged in bay whaling using several sources, and collected additional data from logbooks from a subset of those voyages (Sampling Protocol S1). We estimated total American ship-based catches in New Zealand calving bays by multiplying the likely number of vessels bay-whaling each winter by the average number of right whales reported in the logbooks that we read.

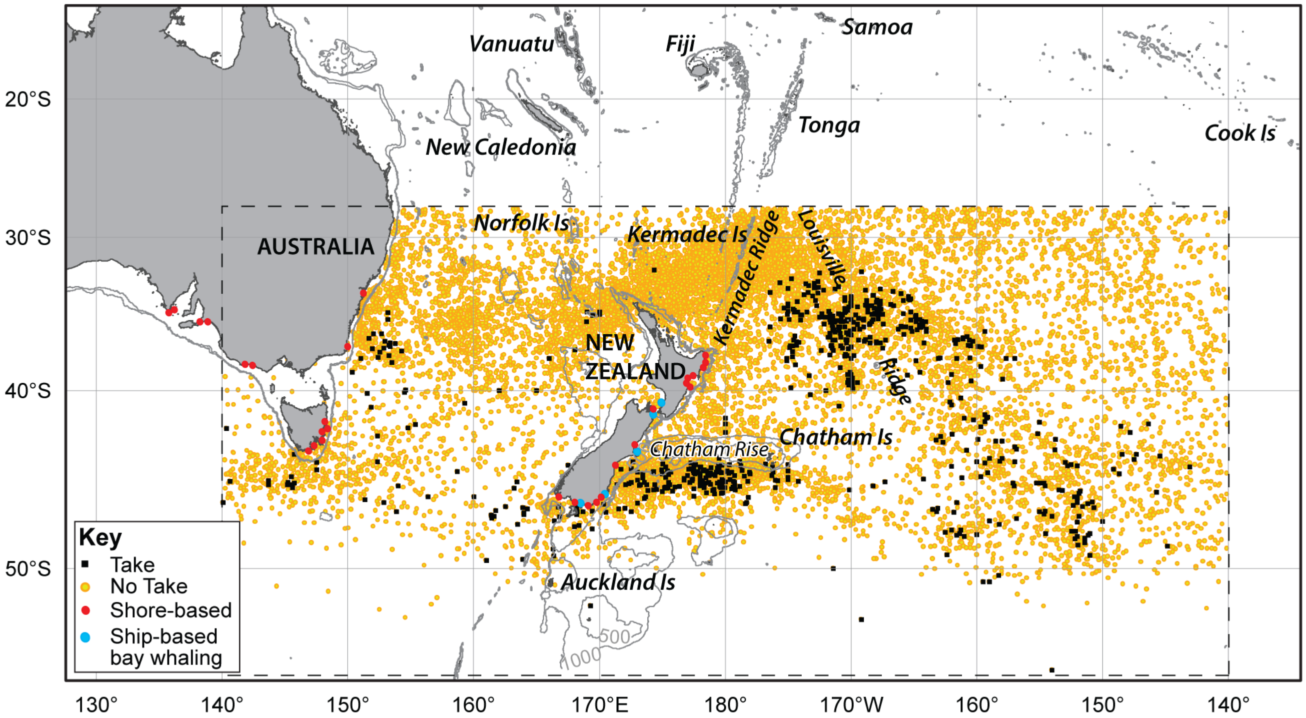

Figure 1. Observations of southern right whales in east Australian and New Zealand waters. New Zealand and east Australia study area showing coastlines, depth contours (500 and 1000 m depth), and the locations of American whaling vessels on days where right whales were taken (black dots) and were not taken (yellow dots), and the locations of winter calving areas where right whales were caught by shore-based whalers (red dots) and by ship-based bay whalers (blue dots).
doi:10.1371/journal.pone.0093789.g001

20th Century Whaling

Tormosov et al. [20] described previously unreported Soviet whaling in New Zealand and east Australian waters, and these data were partitioned between the two regions. No correction for animals struck and lost was applied to these catches because 20th century factory whaling had much lower loss rates [36].

Struck and Lost Factors

In the course of whaling some animals are struck with a harpoon but not ultimately landed, as indicated in some logbooks. Although not all of these struck and lost whales died of their wounds, as shown by whales caught with evidence of having been previously harpooned, an upper bound on the total number of whales removed by whaling can be estimated using the rate at which animals are struck and lost (e.g. [37–38]). The loss rate is thought to vary depending on the conditions of whaling, with whales taken in bays less likely to be lost than those taken offshore, for example, due to a higher likelihood of recovery.

We computed the rate at which right whales were struck but lost using the numbers of whales struck and lost and the number struck and caught reported in logbooks. We treated these data as binomial random variables and estimated the overall proportion (p) of struck animals that were lost, along with its standard error. From this we estimated a loss rate factor (LRF) that can be multiplied by the estimated catches as:

$$LRF = \frac{1}{(1-p)} \qquad (3)$$

The error of this estimate was approximated from the standard error of p using a Taylor Series expansion [39].

Results

We first report estimates of catches of right whales for each of the four fisheries: shore-based whaling, ship-based offshore whaling, ship-based bay whaling, and 20th century whaling. We then report estimates of struck and lost rates, and use those to estimate the total number of right whales removed.

Shore-based Whaling

All primary records used by Dawbin [17] to construct the New Zealand southern right whale coastal catch series were examined and catch series constructed from the information therein (Table 1 and Table S1). Using this information, the primary source for each year of the catch series reported in Dawbin [17] was identified in all but two years (Table S1). For the years 1871–1930, Dawbin [17] used a combination of oil and whalebone export records, in addition to the monetary value of such exports in some years.

Based on the uncertainty in the primary resources, we developed low and high catch scenarios for the New Zealand coastal fishery (Table 1). The low scenario is based on the same primary sources from Dawbin [17] for the most intensive period of the industry: years 1829–1840 [24]. This was considered the low case for these years as it records landings south of Akaroa only, excluding substantial coastal whaling operations in the Cook Strait, Cloudy Bay, and the Kapiti Coast [30]. The low scenario also uses the record with the lower export value derived from the Statistics New Zealand records (oil or baleen) for the years 1854–1930. In contrast, the high case used estimates from McNab [30] for the intensive period of whaling and the higher export values

Table 2. Summary statistics for US offshore whaling voyages departing between from 1837 to 1900, showing the numbers of voyages departing (Voy) and the number of logbooks sampled (Logs).

Dep Year	Voy	Logs	NZ M	NZ N	NZ SE	NZ RW	SE NZ RW	EA M	EAN	EA SE	EA RW	SE EA RW
1837	137	8	0.0	0	0.00	0	0	0.0	0	0.00	0	0
1838	139	6	14.7	3	8.99	2039	1249	0.5	2	0.50	70	70
1839	133	6	29.3	3	8.67	3901	1153	0.0	2	0.00	0	0
1840	149	9	8.3	3	8.33	1242	1242	0.0	1	0.00	0	0
1841	193	11	7.5	8	3.82	1448	737	3.2	5	2.96	618	571
1842	160	9	3.3	4	1.25	520	200	0.5	2	0.50	80	80
1843	193	10	1.3	6	0.61	257	119	0.4	5	0.24	77	47
1844	226	13	2.4	9	1.11	552	250	0.2	6	0.17	38	38
1845	231	17	2.3	15	0.86	539	199	0.2	5	0.20	46	46
1846	133	14	2.3	9	1.86	310	248	0.0	6	0.00	0	0
1847	145	11	0.0	6	0.00	0	0	0.3	4	0.25	36	36
1848	138	9	3.0	5	3.00	414	414	0.0	2	0.00	0	0
1849	100	7	0.0	6	0.00	0	0	0.0	2	0.00	0	0
1850	142	9	0.7	3	0.67	95	95	0.0	0	0.00	0	0
1851	231	6	0.0	4	0.00	0	0	0.0	1	0.00	0	0
1852	121	5	0.0	1	0.00	0	0	0.0	0	0.00	0	0
1853	165	4	0.0	3	0.00	0	0	0.5	2	0.50	83	83
1854	163	4	1.0	1	0.00	163	0	0.0	0	0.00	0	0
1855	134	8	1.8	4	0.75	235	101	0.0	1	0.00	0	0
1856	160	7	0.0	2	0.00	0	0	0.0	0	0.00	0	0
1857	165	5	7.5	2	0.50	1238	83	0.0	1	0.00	0	0
1858	107	5	2.7	3	0.88	285	94	0.0	1	0.00	0	0
1859	82	6	1.0	3	0.00	82	0	1.0	2	1.00	82	82
1860	86	7	0.0	1	0.00	0	0	0.0	0	0.00	0	0
1861	36	2	0.0	1	0.00	0	0	0.0	1	0.00	0	0
1862	70	8	0.0	0	0.00	0	0	0.0	0	0.00	0	0
1863	60	5	0.0	2	0.00	0	0	0.0	1	0.00	0	0
1864	68	5	0.0	0	0.00	0	0	0.0	0	0.00	0	0
1865	97	11	0.0	4	0.00	0	0	2.5	2	2.50	243	243
1866	79	5	0.0	1	0.00	0	0	1.0	1	0.00	79	0
1867	79	3	5.0	2	1.00	395	79	1.0	1	0.00	79	0
1868	75	8	0.0	0	0.00	0	0	0.0	0	0.00	0	0
1869	67	4	0.0	2	0.00	0	0	0.5	2	0.50	34	34
1870	40	1	0.0	0	0.00	0	0	0.0	0	0.00	0	0
1871	43	2	0.0	1	0.00	0	0	0.0	0	0.00	0	0
1872	34	2	0.0	1	0.00	0	0	1.0	1	0.00	34	0
1873	20	0	0.0	0	0.00	0	0	0.0	0	0.00	0	0
1874	21	0	0.0	0	0.00	0	0	0.0	0	0.00	0	0
1875	35	0	0.0	0	0.00	0	0	0.0	0	0.00	0	0
1876	37	1	0.0	0	0.00	0	0	0.0	0	0.00	0	0
1877	46	2	0.0	0	0.00	0	0	0.0	0	0.00	0	0
1878	34	1	0.0	0	0.00	0	0	0.0	0	0.00	0	0
1879	28	2	0.0	0	0.00	0	0	0.0	0	0.00	0	0
1880	36	2	0.0	0	0.00	0	0	0.0	0	0.00	0	0
1881	22	1	0.0	0	0.00	0	0	0.0	0	0.00	0	0
1882	22	2	0.0	0	0.00	0	0	0.0	0	0.00	0	0
1883	16	2	0.0	0	0.00	0	0	0.0	0	0.00	0	0
1884	18	1	0.0	0	0.00	0	0	0.0	0	0.00	0	0

Table 2. Cont.

Dep Year	Voy	Logs	NZ M	NZ N	NZ SE	NZ RW	SE NZ RW	EA M	EAN	EA SE	EA RW	SE EA RW
1885	12	0	0.0	0	0.00	0	0	0.0	0	0.00	0	0
1886	11	1	9.0	1	0.00	99	0	0.0	0	0.00	0	0
1887	13	1	0.0	0	0.00	0	0	0.0	0	0.00	0	0
1888	5	0	0.0	0	0.00	0	0	0.0	0	0.00	0	0
1889	4	0	0.0	0	0.00	0	0	0.0	0	0.00	0	0
1890	4	0	0.0	0	0.00	0	0	0.0	0	0.00	0	0
1891	11	1	0.0	0	0.00	0	0	0.0	0	0.00	0	0
1892	11	1	0.0	0	0.00	0	0	0.0	0	0.00	0	0
1893	10	2	0.0	1	0.00	0	0	0.0	0	0.00	0	0
1894	14	0	0.0	0	0.00	0	0	0.0	0	0.00	0	0
1895	9	2	0.0	0	0.00	0	0	0.0	0	0.00	0	0
1896	5	0	0.0	0	0.00	0	0	0.0	0	0.00	0	0
1897	14	3	0.0	0	0.00	0	0	0.0	0	0.00	0	0
1898	3	0	0.0	0	0.00	0	0	0.0	0	0.00	0	0
1899	10	1	0.0	0	0.00	0	0	0.0	0	0.00	0	0
1900	6	2	0.0	0	0.00	0	0	0.0	0	0.00	0	0
**Total**	**4,858**	**280**				**13,814**					**1,599**	

Separately for New Zealand (NZ) and east Australia (EA), the mean number of right whales taken per sampled logbook (M), and the number of voyages that took right whales in those areas (N), and the standard errors of those means (SE) are shown. Also shown are simple estimates of the total take of right whales in New Zealand (NZ RW) and East Australia (EA RW), and the standard errors of those estimated totals (SE RW).

doi:10.1371/journal.pone.0093789.t002

derived from the Statistics New Zealand records for the years 1853–1930. The totals of the low and high scenarios are 2,703 and 5,104 whales, respectively. The catches of whales from New Zealand at New South Wales are also shown, totaling 2,638. Therefore the total catches for the strict New Zealand low and high scenarios are 5,341 and 7,742, respectively.

The estimated number of right whales killed in the coastal fisheries in east Australia was 13,003 whales: 683 in Victoria, 7,745 in Tasmania and 4,575 in New South Wales (Table 1). These catches were combined with the total New Zealand shore-based catch to estimate the catches for the low and high catch scenarios for east Australia plus New Zealand of 18,344 and 20,745 whales respectively.

Ship-based Offshore Whaling

For American whaling vessels, the number of voyages departing each year varied between 100 to over 200 between 1837 and the late 1850s, before beginning to decline (Table 2). The numbers of logbooks sampled increased from eight for voyages departing in 1837 and peaked at 17 in 1845. The mean number of catches of right whales per voyage varied substantially over those years. The intensity of right whaling was highest for vessels departing their home ports between 1838 and 1842 in both areas, and declined to relatively lower levels from the late 1840s onward. The sum of the estimates of total removals was 13,814 (SE 2,325) and 1,599 (SE 646), for New Zealand and east Australia, respectively (Table 2).

The proportions of American voyages whaling in the study area during each successive season since voyage departure is shown in Table 3. The proportions were relatively constant for the second through the fourth seasons and show that the estimated catches by departure year were in fact taken up to five years after the voyage departure year. We estimated the catches by calendar year from those in Table 2 by assigning the total for each departure year according to the proportions in Table 3.

Du Pasquier [19] reports both catches of individual right whales and landings of right whale products in New Zealand waters for each voyage rather than individual years. He did not have data similar to that in Table 3 but he provided data on the duration of French voyages to New Zealand. We compared that to American vessel voyage duration (Table 4). French voyages were substantially shorter than American voyages, with most lasting three years,

Table 3. The distribution of the year within a voyage (departure year = year 0) that US pelagic vessels whaled in New Zealand and east Australian waters in the 19th century, expressed as the proportion of all voyages that whaled in New Zealand in one or more seasons.

Number of years within a voyage	0	1	2	3	4	5
Proportion	0.007	0.238	0.274	0.262	0.193	0.025

doi:10.1371/journal.pone.0093789.t003

Table 4. The distribution of the number of years duration of US and French pelagic whaling voyages in the 19th century, expressed as a proportion of all voyages with known length.

Country of origin	2 Years	3 Years	4 Years	5 Years	6 or more Years
USA	0.016	0.196	0.359	0.388	0.028
France	0.101	0.624	0.248	0.020	0.007

doi:10.1371/journal.pone.0093789.t004

Table 5. The number of arrivals at ports in the Bay of Islands prior to 1841 for American, British, Australian and French whaling vessels and the proportions of declared cargoes of oil that were only sperm oil, only whale oil and both sperm oil and whale oil summarised by nationality.

Country of Origin	Arrivals	Arrivals with declared oil	Only Sperm Oil	Only Whale Oil	Both Sperm and Whale Oil
America	289	51	0.25	0.18	0.57
Britain	264	21	0.66	0	0.33
Australia	175	26	0.62	0.08	0.31
France	33	17	0.06	0.71	0.23

doi:10.1371/journal.pone.0093789.t005

compared to five years for American voyages. We estimate French catches by departure year by allocating them equally to two, three and four years after departure, because of the relative consistency of the American proportions for voyage years three, four and five.

Although Dawbin [17] had no estimates for catches of right whales by vessels from other than France and the US, he suggested that landings by British vessels should be examined, that landings by Australian vessels were likely included in Australian landings statistics, and that other nations' ship were few. To examine this, we identified in Richards and Chisholm [35] 755 arrivals of whaling ships making port in the Bay of Islands prior to 1840. There were only seven, five and two arrivals from Germany, Canada and Portugal, respectively, confirming Dawbin's [17] conclusion that right whaling by these nations was minimal. The proportions of arriving ships with only sperm oil, only whale oil, and mixed sperm and whale oil varied among American, British, Australian and French nationalities (Table 5). For example, British and Australian vessels reported only sperm oil for 66% and 62% of arrivals, respectively, while American and French vessels reported only sperm oil much less frequently, 25% and 6% of arrivals, respectively. The numbers of port visits suggest that there were substantial numbers of vessels from Britain and Australia whaling in this area, and at least some of the time they pursued right whales. Although we have no estimates of right whales by other nations, the fact that other nationalities focused more on sperm than right whales suggests that any such catches would have been fewer than those by American and French vessels.

Ship-based Bay Whaling

We identified over 300 vessel-seasons that were in a geographical position to pursue bay whaling in New Zealand. Of these we judged that 106 and 59 were highly likely and possibly bay whaling, respectively, for a total of 165 vessels (See supplementary material: Sampling Protocol S1). We obtained the number of whales caught, and those struck and lost from some of these logbooks (Table 6). The logbook for the *Jasper* (Voyage Identification Number 7413 from [33]) reported catches for two other vessels it was working with, and as we were unable to assign whales to individual vessels, we assigned each vessel one third of the total reported. Four of the winter catches were reported in barrels of whale oil, and we divided those by 40, the average number of barrels per whale reported previously for New Zealand bay whaling [17]. This value was not statistically different from the reported barrels of oil reported for seven whales in the *Courier* logbook (mean 48 barrels, SE 9.8: Voyage Identification Number 3448 from [33]). The mean number of barrels obtained per whale during bay whaling was lower than the mean during offshore whaling (81 barrels, AOWL), which is consistent with calves being reportedly taken seven times in the bay whaling logbooks (Table 6).

The number of right whales caught per winter vessel-season for the 14 seasons averaged 23.3 (SE 3.72), but varied over the years (Table 6). For example, the average catches for the three vessels sampled in 1839 was significantly less than for the 12 vessels sampled prior to 1839 ($p<0.01$). We multiplied the number of vessel-seasons for each year that were definitely and possibly bay whaling by the average number caught per season to obtain estimates of the total right whales from 1834 to 1841. The total numbers caught over the period for the two cases were 2,404 (SE 173.5) and 3,781 (SE 274.4) for the highly likely and highly likely plus possibly bay whaling vessels, respectively (Table 7).

20th Century Whaling

Soviet whaling catches between 1963–1966 in the New Zealand sub-Antarctic islands and to the west and north totalled 294 animals. The majority were taken near the Auckland Islands [20,40]. A further 78 whales were taken south of Tasmania at 47°S and 150°E in 1969/1970 [20].

Struck and Lost Rates

Using information from the AOWL data and additional data obtained from bay whaling vessels in New Zealand calving bays (Table 8), we estimated struck and lost factors for three situations: north and south of the equator and in New Zealand whaling bays (Table 8). The proportion of right whales that were struck and lost was significantly higher ($p<0.01$) in the northern (0.50, SE 0.12) than in the southern hemisphere (0.31, SE 0.05). The proportion of right whales that were struck and lost in calving bays (0.21, SE 0.05) was significantly lower than the Southern Hemisphere offshore proportion, which is consistent with the view that the calmer and more constrained conditions in bays would facilitate catching whales [37–38].

Total 19th and 20th Century Catches and Removals

We partitioned the estimated catches in Tables 1, 2, and 7 and in Dawbin's [17] estimates of French ship-based catches among three fisheries: shore-based whaling, ship-based offshore whaling and ship-based bay whaling. We assumed that the reported Soviet removals were all of the modern 20th century catches of right whales.

Low and high scenarios for shore-based catches for New Zealand and for New Zealand plus east Australia together were summarised from Table 1. The ship-based offshore catches for New Zealand and for east Australian waters were from Table 3 (column NZ RW and column EA NZ RW) and proportions of Dawbin's French ship-based estimates. Those proportions were estimates as the fractions of American ship-based catches in New Zealand and in east Australian waters (Table 2). Within New Zealand, the estimated French ship-based catches were then

Table 6. The number of southern right whales (*Eubalaena australis*) caught, struck and lost (S&L) and calves caught during the austral winter, mentioned in a sample of logbooks of US voyage (Sampling Protocol S1), and for four vessels numbers of barrels of right whale oil obtained during those months reported in a logbook or by McNab [30], with location of logbooks shown identified by VID (Voyage Identification Number from [33]).

Year	Vessel	VID	Bay	Caught	S&L	Calves	Barrels
1836	*Columbus*	3061	Otago				1600
1836	*Erie*	4590	Cloudy Bay	20.3[A]			
1836	*Friendship*	5330	Port Cooper				1800
1836	*Gratitude*	3003	Bluff Harbour				1050
1836	*Jasper*	7413	Cloudy Bay	20.3[A]			
1836	*Martha*	9148	Otago				1700
1836	*Mary Mitchell*	9384	Otago	22	4		
1836	*South Boston*	13272	Cloudy Bay	20.3[A,B]			
1837	*Courier*	3448	Bluff Harbour	33	16	2	
1838	*Alexander Barclay*	512	Bluff Harbour	23	5	1	
1838	*Columbus*	3060	Otago	39	0	2	
1838	*Friendship*	5331	Otago	3.5	3		
1839	*Amethyst*	875	Bluff Harbour	7	4		
1839	*China*	2846	Kapiti	2	1	1	
1839	*Samuel Robertson*	12808	Cloudy Bay	6	3	1	

[A]From logbook of the *Jasper*, reporting a total of 61 whales taken by three mated vessels.
[B]Indicates the logbook reports other whalers taking cow and calf pairs.
doi:10.1371/journal.pone.0093789.t006

partitioned into the offshore and bay whaling fisheries according to the fraction of American ship-based estimates in offshore waters and in bays. In making this calculation, we used the mean of the high and low estimates for American ship-based bay whaling because the differences in the proportions were minimal.

We estimated total removals by multiplying the estimates of catches (Table 9; shown by year in Table S2) by the appropriate loss rate correction factors (Table 8) for each fishery: 1.27 for bay and shore whaling and 1.45 for offshore fisheries. The total removals from New Zealand waters were between a low of 34,002 and a high of 38,800, while the total removals from New Zealand and east Australia waters combined were between a low of 53,145 and a high of 57,958 right whales. The annual estimated catches by fishery varied greatly over time (Figure 2). We have not reported the statistical uncertainties of these totals because estimates of sampling variances are not available for the estimates of shore-based whaling, but by giving low and high scenarios for these fisheries we attempted to investigate the variance.

Table 7. Numbers of American whaling vessels highly likely (H) and possibly (P) bay whaling in each winter in New Zealand, and estimates of numbers of southern right whales (*Eubalaena australis*) removed (E), with standard errors (SE).

Year	H	H+P	E(H)	SE(H)	E(H+P)	SE(H+P)
1834	1	1	23	3.7	23	3.7
1835	1	1	23	3.7	23	3.7
1836	18	19	420	67.0	444	70.7
1837	12	16	280	44.6	373	59.5
1838	22	31	514	81.9	724	115.3
1839	31	40	724	115.3	934	148.8
1840	16	47	373	59.5	1097	174.9
1841	2	7	47	7.4	163	26.0
**Totals**	**103**	**162**	**2404**	**173.5**	**3782**	**274.4**

doi:10.1371/journal.pone.0093789.t007

Discussion

Our estimates of total removals of right whales from New Zealand and eastern Australia over the 19th and 20th centuries are substantially higher than previous estimates, increasing from the 26,000 estimated by Dawbin [17] to between 53,000 and 58,000 estimated here. Right whaling was pursued by whalers from several countries over the two centuries, however, 82% of removals were concentrated over the two decades between 1830 and 1849. Whaling was most intense over the decade from 1835 to 1844, accounting for 66% of the removals, a pattern similar to that found by Dawbin [17]. Right whaling continued in New Zealand

Table 8. Estimates of the proportion of right whales (*Eubalaena spp.*) struck but lost (P) with standard error (SE(P)) by US whalers in the northern hemisphere (North) and the southern hemisphere (South) and in New Zealand bays (New Zealand) during the 19th century.

Region	Strikes	P	SE(P)	LRF	SE(LRF)
North	302	0.50	0.029	2.00	0.115
South	328	0.31	0.026	1.45	0.054
New Zealand	171.5	0.21	0.031	1.27	0.050

Also shown are the corresponding loss rate factors (LRF, defined as 1/(1-P)) with standard errors (SE(LRF)).
doi:10.1371/journal.pone.0093789.t008

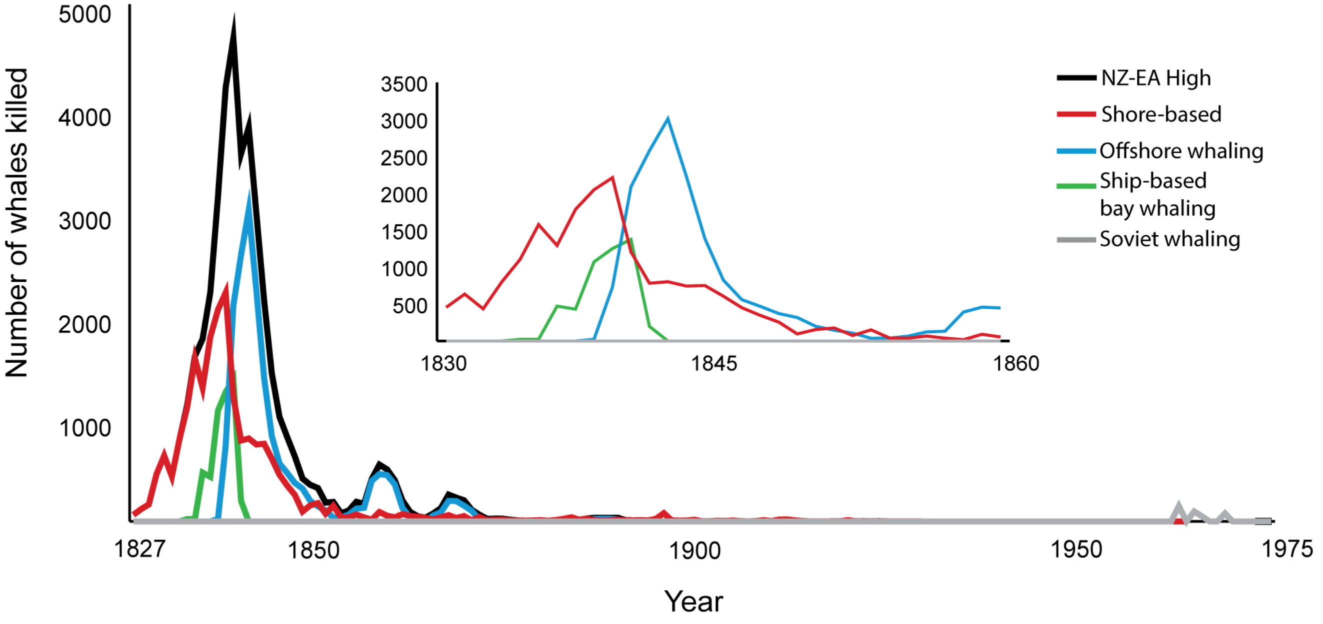

Figure 2. Catches of right whales (*Eubalaena australis*) around New Zealand and east Australia. The catches of southern right whales (number of whales killed) for the New Zealand plus east Australia high scenario are shown for the time period 1827 to 1975. No adjustment for struck and lost rate has been made to these catches.
doi:10.1371/journal.pone.0093789.g002

over the remainder of the 19th century and even in the 20th century. The result was that southern rights were rare around the New Zealand mainland for most of the 19th century and were not seen at all for nearly four decades of the 20th century [41]. The effect of the initial removals was to drastically reduce the abundance of right whales. Due to the resulting low abundance, the relatively limited removals during the late 19th and 20th century were a significant contributing factor to the failure of right whales to recover in these waters for over 100 years.

The intensity of the shore, bay and offshore fisheries varied over time. Shore-based whaling declined abruptly and ship-based bay whaling by both French and American whalers ended around 1841, coincident with the British claims on New Zealand [42] and the discovery of the northwest grounds off the Alaskan coast [43]. Offshore whaling increased as bay whaling declined, and continued for some years. The ongoing low level of whaling, culminating in the Soviet catches in the late 20th century, was sufficient to keep the population at apparent low levels (Figure 2).

Table 9. Estimates of total catches and removals of southern right whales (*Eubalaena australis*) by fishery and scenario for New Zealand and east Australia.

**Fishery**	**New Zealand - Low**			**New Zealand - High**		
	**Catches**	**S&L**	**Removals**	**Catches**	**S&L**	**Removals**
Shore-based whaling	5,341	1.27	6,783	7,742	1.27	9,832
Ship-based offshore whaling	16,463	1.45	23,871	16,463	1.45	23,871
Ship-based bay whaling	2,404	1.27	3,053	3782	1.27	4,802
Modern (Soviet) whaling	294	N/A	294	294	N/A	294
**Total**	**24,502**		**34,002**	**29,342**		**38,800**
**Fishery**	**New Zealand - Low plus east Australia**			**New Zealand - High plus east Australia**		
	**Catches**	**S&L**	**Removals**	**Catches**	**S&L**	**Removals**
Shore-based whaling	18,344	1.27	23,297	20745	1.27	26,346
Ship-based offshore whaling	18,223	1.45	26,423	18233	1.45	26,438
Ship-based bay whaling	2404	1.27	3,053	3782	1.27	4,802
Modern (Soviet) whaling	372	N/A	372	372	N/A	372
**Total**	**39,343**		**53,145**	**43,122**		**57,958**

The struck and lost rate (S&L) applied to each fishery is shown and was used as a multiplier of the catches to calculate removals between 1827 and 1975. Four scenarios are considered: low and high for New Zealand and New Zealand plus east Australia.
doi:10.1371/journal.pone.0093789.t009

These new estimates are a substantial improvement over previous estimates because we drew on substantial new data, particularly for the American pelagic and bay fisheries. We also accounted better for the distribution of catches over the several years of American and French voyages and we estimated total removals based on new information on whales that were struck but not landed.

Remaining uncertainties

Substantial uncertainties remain, however, and we explored the potential magnitude of two of these. One is the difference in the estimates of New Zealand and east Australian shore-based whaling by Dawbin [17] and McNab [30]. The other is the uncertainty about the numbers of American vessels participating in the winter whaling in calving bays around New Zealand. The difference in the low and high estimates based on those uncertainties is less than 2,500 whales each, <5% percent of the total removals.

Unexplored uncertainties include those that likely biased the catch series downwards, such as uncertainty in the struck and loss rate, using export rather than landing records, the killing of calves as part of the fishery, and not accounting for whaling vessels of other nationalities. There are also uncertainties that will have an unknown effect on the catch series, such as the relatively low number of extant logbooks available for the study.

Although we have now accounted for struck and loss rates, the available data are sparse: additional reading of as yet unread US voyage logbooks would help here. The proportion of the struck but lost whales that survived is unknown so there is also likely to be an upward bias in our estimates; it is not apparent how to address this uncertainty.

For shore-based whaling, the later part of the catch series is based on export records [31] as no catch data were located. These estimates were likely affected to an unknown degree by local consumption and the possibility the year of export was not the year the whale was caught. We were also unable to directly assess the east Australian shore-based catch series, which is a significant part of the catches, and instead had to rely on Dawbin's [17] records.

Futhermore, much anecdotal evidence was found that suggests coastal whalers routinely targeted cow-calf pairs, for example, Sherrin [21] states that a large proportion of the catch was calves. It was routine to kill the calf in order to secure the larger, more valuable right whale cow [21,30,32]. Our reading of American vessel-based bay whaling suggested that calves were taken frequently (7 of 15 read log books record this). This is consistent with the average barrels of oil obtained per whale indicated in one logbook (48 barrels per whale) compared to the average of 81 barrels per whale for offshore whaling indicated by the AOWL data. Similarly, this is consistent with Dawbin's [17] observation that the coastal industry had a lower yield of 4.18 tuns per whale, compared with 6 tuns per whale for the pelagic fishery. It is reasonable to suppose the lower yield is due to the smaller volume of oil extracted from the young whales. For example, Sherrin [21] states that one-year old calves produced approximately 4 tuns of oil, and given the fishery targeted the calving grounds, many calves and yearlings would have been younger and smaller whales. In addition, the size and condition of females would have decreased over the wintering season due to weight loss from lactation.

For ship-based whaling, we assumed that there were no right whale removals by ships from Britain and Australia, as well as Germany, Canada, and Portugal. While port arrival records suggest that right whales were less frequently taken by British and Australian vessels, it is likely that the Australian based landings were accounted for at least in part in the landing statistics described under shore-based whaling. The available data do not allow separate estimates to be made for either the Australian ship-based or the British ship-based whaling. Information on how many voyages such vessels made might be found in other vessel arrival and departure data in different ports. Further, additional information on the rate at which right whales were taken might be found in logbooks from such voyages if these can be located. For example, a number of logbooks from the 19th century Tasmania ship-based offshore fishery are known to exist [44], and examination of these would be useful, especially for the number, species and spatial distribution of catches, oil yield per whale, and potentially struck and lost rates. The lack of inclusion of these whaling fleets will bias the estimate low.

For American whaling, our logbook-based estimates overcame some of the uncertainties in Dawbin's [17] estimates. However, the sample of logbooks for both offshore whaling and bay whaling was sparse and the mean catches of right whales by vessels departing in the earlier years varied substantially. The low sampling intensity and rapid changes in whaling intensity mean the reliability of the annual estimates is relatively low. For example, the apparent post-bonanza pulses of catches in the mid-1800s are based on few samples, and alternative methods of pooling those data over time and alternate ways of assigning the departure year estimates to calendar year would likely result in a more protracted pattern of whaling over this period.

To determine priorities for additional historical study, it would be useful to examine the biological effects of this whaling using population modeling approaches aimed at regional rather than circumpolar assessment [15,45]. This would allow one to evaluate the potential value of improved estimates of removals for our understanding of right whales in this region.

Conclusion

Our results confirm a pattern of increasingly intense whaling and rapid depletion of populations of right whales over time. In the North Atlantic, right whaling spread out of the Bay of Biscay beginning around 1000 AD, sequentially depleting populations in the eastern and then western North Atlantic up to around 1850 (Table 2.1, [46]), nearly annihilating that species over several centuries. In the present case, beginning in the 1830s, substantial right whaling persisted for only two decades in New Zealand. Subsequently in the North Pacific, right whaling persisted for only the decade of the 1840s [43]. In the North and South Pacific in the 1830s and 1840s the AOWL data reveal that some of the same vessels pursued right whales in both hemispheres during the same voyage, seasonally shifting from one to the other, as previously described [22]. Despite this obvious pattern of intense and increasingly unsustainable right whaling, no limits were placed on hunting right whales until the 1930s. But even after that, the value of the animals prompted commercial whalers to again illegally hunt right whales in the mid-20th century, both near New Zealand and elsewhere.

Similar patterns of short duration offshore vessel whaling have been seen for other large whales. Thus gray whales in the North Pacific were depleted in 25 years, between 1845 and 1870 [37]. Humpback whales in the North Atlantic were depleted in 60 years, between 1850 and 1910 [38]. Blue whales were depleted around Iceland in a decade, between 1904 and 1914 [47–48].

Such rapid changes in abundance are not linked to the oft mentioned 'shifting baseline syndrome' [49] because the changes occurred within the working life of both whalers and their managers. Further, these changes are so rapid that their effects on whaling activities have been made evident since at least the middle of the 19th century in books and maps. The baselines have been as

evident as the willingness to continue to pursue whaling to industrial extinction and whales to near extinction [50].

Supporting Information

Sampling Protocol S1 Methods for obtaining American logbook data for New Zealand calving bays.
(DOC)

Table S1 Estimated shore-based whaling catches, listed as number of southern right whales, with primary source listed in column to right of catch for catches at New South Wales that were from New Zealand (NSW-NZ) and two estimated coastal catch series for New Zealand (NZ-Low, NZ-High), reflecting different selections of primary sources.
(DOCX)

Table S2 Catches by fishery by year for the New Zealand and east Australian southern right whale catch series. Shore.NZ.L: catches of the New Zealand coastal shore-based fishery under the low scenario; Shore.NZ.H: catches of the New Zealand shore-based fishery under the high scenario; Shore.EA: catches of the east Australian shore-based fishery from Dawbin [17]; Off.NZ: catches of the New Zealand ship-based offshore fishery; Off.EA: catches of the east Australian ship-based offshore fishery; Bay.L: catches of the New Zealand ship-based bay whaling fishery under the low scenario; Bay.H: catches of the New Zealand ship-based bay whaling fishery under the high scenario; Soviet.EA: modern Soviet whaling in east Australian waters from Tormosov et al. [20]; Soviet.NZ: modern Soviet whaling in New Zealand waters from Tormosov et al. [20].
(XLSX)

Acknowledgments

The authors would like to thank Janet Dawbin and Peter Gill for their assistance with the catch series reconstruction, and Macquarie Library for access to source materials. We are also indebted to an anonymous reviewer whose comments greatly improved the manuscript and C. Scott Baker, Alison MacDiarmid and Rohan Currey for comments on earlier versions of manuscript. We thank Erika Mackay for help with the Figures. Numerous people assisted in assembling and extracting the American logbook data, including R. Reeves, J. Lund, E. Josephson, R. Richards, J. Bannister, D. Chatwin, O. Hamilton and R. Constantine who have laboriously extracted information from logbooks. We are also indebted to the institutions holding the whaling voyage logbooks, especially the Kendall Institute of the New Bedford Whaling Museum in New Bedford, Massachusetts; the Blunt White Library, the Mystic Seaport, Mystic, Connecticut, several others in the New England region, and the Pacific Manuscripts Bureau for access to copies of several logbooks examined.

Author Contributions

Conceived and designed the experiments: EC TS JJ DP. Performed the experiments: TS EC. Analyzed the data: TS JJ DP. Contributed reagents/materials/analysis tools: TS EC JJ DP. Wrote the paper: EC TS JJ DP.

References

1. Reeves RR, Smith T (2007) A Taxonomy of World Whaling: operations, eras and data sources. In: Estes JA, Brownell R, DeMaster DP, Doak DF, Williams TM, editors. Whales, whaling and ocean ecosystems. Berkley, CA: University of California Press.
2. IWC (1986) Right whales: Past and Present Status. Report of the International Whaling Commission 44: 146–152.
3. Wade P, Kennedy A, LeDuc RG, Barlow J, Carretta J, et al. (2011) The world's smallest whale population? Biology Letters 7: 83–85.
4. Fujiwara M, Caswell H (2001) Demography of the endangered North Atlantic right whale. Nature 414: 537–541.
5. Josephson E, Smith T, Reeves RR (2008) Historical distribution of right whales in the North Pacific. Fish and Fisheries 9: 155–168.
6. Brandão A, Best P, Butterworth D (2010) Estimates of demographic parameters for southern right whales off South Africa from survey data 1979 to 2006. Unpublished report (SC/62/BRG30) presented to the Scientific Committee of the International Whaling Commission.Cambridge, UK
7. Reilly SB, Bannister JL, Best P, Brown MW, Brownell R, et al. (2008) *Eubalaena australis* (Chile-Peru subpopulation). IUCN 2009 IUCN Red List of Threatened Species Version 20092. Available: http://www.iucn.redlist.org.
8. Patenaude NJ, Portway V, Schaeff C, Bannister JL, Best P, et al. (2007) Mitochondrial DNA diversity and population structure among southern right whales (*Eubalaena australis*). Journal of Heredity 98: 147–157.
9. IWC (2001) Report of the workshop on the comprehensive assessment of right whales. Journal of Cetacean Research and Management Special Issue 2: 1–60.
10. Richards R (2002) Southern right whales: a reassessment of their former distribution and migration routes in New Zealand waters, including the Kermadec grounds. Journal of the Royal Society of New Zealand 32: 355–377.
11. Carroll EL, Rayment W, Alexander AE, Baker CS, Patenaude NJ, et al. (2014) Reestablishment of former wintering grounds by the New Zealand southern right whales. Marine Mammal Science 30: 206–220.
12. Carroll EL, Childerhouse S, Fewster R, Patenaude NJ, Steel D, et al. (2013) Accounting for female reproductive cycles in a superpopulation capture recapture framework. Ecological Applications 23: 1677–1690.
13. Carroll EL, Patenaude NJ, Alexander AM, Steel D, Harcourt R, et al. (2011) Population structure and individual movement of southern right whales around New Zealand and Australia. Marine Ecology Progress Series 432: 257–268.
14. Bannister JL (2011) Population trend in right whales off southern Australia 1993–2010. Unpublished report (SC/S11/RW10) presented to the International Whaling Commission Workshop on Southern right whales, 13–16 September 2011, Buenos Aires, Argentina.
15. IWC (2012) Report of the workshop on the assessment of southern right whales, Buenos Aires, Argentinal 13–16 September 2011. Unpublished report (SC/64/Rep5) presented to the Scientific Committee of the International Whaling Commission.Cambridge, UK
16. Kato H, Matsuoka K, Nishiwaki S, Bannister JL (2007) Distributions of abundance of pygmy blue whales and southern right whales in waters off southern coast of Australia based on data from the Japan/IWC blue whale cruise 1996–96. Unpublished report (SC/59/SH10) presented to the Scientific Committee of the IWC, Cambridge, UK.
17. Dawbin W (1986) Right whales caught in waters around south eastern Australia and NZ during the nineteenth and early twentieth centuries. Report of the International Whaling Commission Special Issue 10: 261–268.
18. Starbuck A (1878) History of the American whale fishery from its earliest inception to the year 1876. Washington: United States Commission of Fish and Fisheries. Part IV. Report of the Commissioner for 1875–1876. Appendix A. Sea Fisheries.
19. Du Pasquier T (1986) Catch history of French right whaling mainly in the South Atlantic. Report of the International Whaling Commission Special Issue 10: 268–274.
20. Tormosov D, Mikhaliev Y, Best P, Zemsky V, Sekiguchi M, et al. (1998) Soviet catches of Southern right whales *Eubalaena australis* 1951–1971. Biological Conservation 86: 185–197.
21. Sherrin R (1886) Handbook of the fishes of New Zealand. Wilsons & Horton.Auckland, NZ
22. Bannister JL (1986) Notes on nineteenth century catches of southern right whales off the southern coasts of Western Australia. Report of the International Whaling Commission Special Issue 10: 255–259.
23. Dawbin W, Dawbin J (1985) Historical documents relating to right whaling in Australian and New Zealand waters during the nineteenth and twentieth centuries. Report to the Australian National Parks and Wildlife Service.Can-Canberra
24. Great Britain Parliamentary Paper (1846 (337)) Communcations between Lord Stanley and Govenor Fitzroy and Lieutenant Governor Grey, relative to New Zealand.
25. Great Britain Parliamentary Paper (1850 (1135)) Report from the Episcopal and Capitular Revenues Commissioners.
26. Great Britain Colonial Office (1853) Blue book of statistics, 1841–1853. Canberra: Australian Joint Copying Project, National Library of Australia.
27. McNab R (1907) Murihiku and the Southern Islands. Invercargill: William Smith.
28. McNab R (1908–1914) Historical records of New Zealand. Wellington: Government Printer.
29. McNab R (1909) Discovery of the Islands. The subantarctic islands of New Zealand Volume I. C.Wellington Philosophical Institute of Canterbury.
30. McNab R (1913) The old whaling days: A history of southern New Zealand from 1830 to 1840. Christchurch: Whitcombe & Tombs.
31. Statistics New Zealand (1900) Statistics of New Zealand 1854–1900. Wellington: Government Printer.
32. Wakefield EJ (1848) The handbook for New Zealand. London: John W. Parker.
33. Lund JN, Josephson E, Reeves RR, Smith T (2010) American Offshore Whaling Voyages: 1667–1927. Volume I: Voyages by Vessel; Volume II: Voyages by

Master. Old Dartmouth Historical Society – New Bedford Whaling Museum.-.New Bedford Massachusetts.
34. Smith TD, Reeves RR, Josephson E, Lund JN (2012) Spatial and seasonal distribution of American whaling and whales in the age of sail. PLoS ONE 7: e34905. doi:34910.31371/journal.pone.0034905.
35. Richards R, Chisholm J (1992) Bay of Islands shipping arrivals and departures. Canberra: Roebuck Society.
36. Clapham P, Baker CS (2002) Modern whaling. In: Perrin WF, Wursig B, Thewissen JGM, editors. Encyclopaedia of Marine Mammals. New York: Academic Press. pp. 1328–1332.
37. Reeves RR, Smith T, Lund JN, Lebo S, Josephson E (2010) Nineteenth century ship-based catches of gray whales, *Eschirichtius robustus*, in the eastern North Pacific. Marine Fisheries Review 72: 26–65.
38. Smith T, Reeves RR (2010) Historical catches of humpback whales, *Megaptera novaeangliae*, in the North Atlantic Ocean: estimates of landings and removals. Marine Fisheries Review 72: 1–43.
39. Seber G (1973) Estimation of animal abundance and related parameters. London: Griffin.
40. Patenaude NJ (2002) Demographic and genetic status of right whales at the Auckland Islands, New Zealand. PhD thesis. Auckland: University of Auckland, Auckland, New Zealand.
41. Gaskin DE (1964) Return of the southern right whale (*Eubalaena australis* Desm.) to New Zealand waters, 1963. Tuatara 12: 115–118.
42. Morton H (1982) The whale's wake. Honolulu: University of Hawaii Press.
43. Josephson E, Smith T, Reeves RR (2008) Depletion within a decade: the American 19th-century North Pacific right whale fishery. In: Starkey A, Holm P, Barnard M, editors. Oceans Past: Management insights from the History of Marine Animal Populations. London: Earthscan.
44. Richards R (2009) Sir William Crowther's whaling collection in Hobart in a global context. The Great Circle 31: 59–65.
45. Jackson JA, Patenaude NJ, Carroll EL, Baker CS (2008) How few whales were there after whaling? Inference from contemporary mtDNA diversity. Molecular Ecology 17: 236–251.
46. Reeves RR, Smith TD (2006) Near annilization of a species: Righ whales in the North Altantic. In: Kraus SD, Rolland RM, editors. The Urban Whale: Harvard University Press.
47. Hjort J (1933) Whales and whaling. Hvalradets Skrifter 7: 7–29.
48. Smith T (1994) Scaling Fisheries. Cambridge: Cambridge University Press.
49. Papworth S, Rist J, Coad L, Milner-Gulland E (2008) Evidence for shifting baseline syndrome in conservation. Conservation Letters 2: 93–100.
50. Dorsey K (2014) Whales and Nations: Environmental diplomacy on the high seas. Seattle: University of Washington Press.
